# Histone Chaperone Asf1 Plays an Essential Role in Maintaining Genomic Stability in Fission Yeast

**DOI:** 10.1371/journal.pone.0030472

**Published:** 2012-01-26

**Authors:** Katsuhiro Tanae, Tomitaka Horiuchi, Yuzy Matsuo, Satoshi Katayama, Makoto Kawamukai

**Affiliations:** Department of Life Science and Biotechnology, Faculty of Life and Environmental Science, Shimane University, Matsue, Japan; Oregon State University, United States of America

## Abstract

The histone H3-H4 chaperone Asf1 is involved in chromatin assembly (or disassembly), histone exchange, regulation of transcription, and chromatin silencing in several organisms. To investigate the essential functions of Asf1 in *Schizosaccharomyces pombe*, *asf1-ts* mutants were constructed by random mutagenesis using PCR. One mutant (*asf1-33*(ts)) was mated with mutants in 77 different kinase genes to identify synthetic lethal combinations. The *asf1-33* mutant required the DNA damage checkpoint factors Chk1 and Rad3 for its survival at the restrictive temperature. Chk1, but not Cds1, was phosphorylated in the *asf1-33* mutant at the restrictive temperature, indicating that the DNA damage checkpoint was activated in the *asf1-33* mutant. DNA damage occured in the *asf1-33* mutant, with degradation of the chromosomal DNA observed through pulse-field gel electrophoresis and the formation of Rad22 foci. Sensitivity to micrococcal nuclease in the *asf1-33* mutant was increased compared to the *asf1^+^* strain at the restrictive temperature, suggesting that *asf1* mutations also caused a defect in overall chromatin structure. The Asf1-33 mutant protein was mislocalized and incapable of binding histones. Furthermore, histone H3 levels at the centromeric outer repeat region were decreased in the *asf1-33* mutant and heterochromatin structure was impaired. Finally, *sim3*, which encodes a CenH3 histone chaperone, was identified as a strong suppressor of the *asf1-33* mutant. Taken together, these results clearly indicate that Asf1 plays an essential role in maintaining genomic stability in *S. pombe*.

## Introduction

The nucleosome is the basic unit of most eukaryotic chromatin. It consists of four core histones (H2A, H2B, H3, and H4) with DNA wrapped around them [1]. Chromatin is highly dynamic and often changes its structure. For example, when a gene is expressed in response to signaling, histones at the promoter regions are evicted during transcription [2]. In addition, histones at sites of DNA damage are removed and newly synthesized histones are incorporated into repaired sites after completion of DNA repair. These structural changes in chromatin are mediated in part by histone chaperones.

Histone chaperones mediate chromatin assembly or disassembly through binding to histone proteins [3], [4]. Asf1 (anti-silencing function1) is a histone H3/H4 chaperone that functions in DNA replication-dependent and -independent chromatin assembly together with other histone chaperones such as CAF1 (Chromatin assembly factor1) and HIRA (Histone interacting protein A) [5]. *asf1* was originally identified as a gene that derepressed the silent mating type loci when overexpressed in *Saccharomyces cerevisiae* [6].

Asf1 was biochemically purified as a chromatin assembly factor from *Drosophilla melanogaster* embryo extracts [7]. It is highly conserved across many species from yeasts to metazoans. During DNA replication in human cells, Asf1 binds to MCM (Mini Chromosome Maintenance) helicase, and evicts old histones H3/H4 from the front of the replication forks [8], and may transfer them to CAF1. CAF1 then deposits histones H3/H4 onto newly synthesized DNA strands. During transcription, Asf1 evicts histones H3/H4 from the promoter regions of genes [9], enabling transcription factors or RNA polymerases to function on DNA strands.

Three-dimensional structures of Asf1 from *S. cerevisiae*, *Schizosaccharomyces pombe* and humans have been resolved, and the co-crystal structure of *S. cerevisiae* Asf1p or human ASF1a (CIA-I) with the histones H3/H4 dimer has also been resolved [10], [11]. These ASF1 structures were all similar and the primary binding site between ASF1 and histones H3/H4 was located in the ASF1 ß1- and ß10-strands and the α3- and α2-helix of H3. The histones H3/H4 tetramer-disrupting activity found in ASF1a supports the nucleosome assembly/disassembly role of Asf1 [10], [11].

In *S. cerevisiae*, many histone chaperones including Asf1, CAF1 and HIRA are cooperatively involved in chromatin structure change. *S. cerevisiae* Asf1 has been shown to be involved in DNA replication-dependent or -independent nucleosome assembly, histone acetylation, histone exchange, regulation of transcription, and chromatin silencing [12], [13], [14], [15], [16], [17]. Although Asf1 is dispensable in *S. cerevisiae*, *asf1* and its orthologs are essential for survival in *S. pombe*, *D. melanogaster* and chicken DT-40 cells [18], [19], [20], [21]. This may reflect the capacity of histone chaperones in *S. cerevisiae* to replace the function of Asf1. Extensive efforts have been made to understand the role of Asf1 in *S. cerevisiae* but the analysis of *asf1* in other species including *S. pombe* is still limited [21], [22]. Analysis in *S. pombe* should provide important information on the essential role of Asf1 in cells as a model organism.

To better understand the role of *asf1* in *S. pombe*, we created an *S. pombe asf1* temperature sensitive mutant (*asf1-33*) and showed that the mutation caused double strand breaks in DNA, increased the sensitivity of chromatin DNA to micrococcal nuclease (MNase), and subsequently activated the DNA damage checkpoint pathway. The defects in chromatin structure in the *asf1-33* mutant at the restrictive temperature caused DNA damage, which induced the cell cycle checkpoint response mediated by Chk1, indicating that *asf1* is essential for the maintenance of genomic stability in fission yeast. We also found genetic evidence suggesting functional similarity between Asf1 and a Cen H3 histone chaperone, Sim3.

## Materials and Methods

### Yeast strains and general methods

The fission yeast strains examined in this study are listed in Table 1. Each strain was cultured in YES medium (0.5% yeast extract, 3% glucose, 225 mg/liter adenine, histidine, leucine, uracil, and lysine hydrochloride) or EMM2 medium. Nitrogen-free EMM2 medium was used to mate *h* ^−^ and *h* ^+^ strains. General methods using *S. pombe* were performed as described previously [23].

**Table 1:** Searching for protein kinase required for survival and cell elongation of *asf1-33* mutant.

gene	phenotype	gene	phenotype	gene	phenotype	gene	phenotype
bub1	EV	ppk10	EI	hhp1	EV	ppk21	EI
ssp2	EV	ppk9	EV	cmk1	EI	ppk38	EV
ppk36	EV	ppk8	EI	cek1	EV	ppk1	EV
ppk35	EV	sck2	EI	wee1	NV	ppk6	EV
gad8	EV	hri2	NV	pit1	EI	cki2	EI
oca2	EV	hri1	EV	mkh1	EV	pef1	NV
gsk31	EV	ppk15	EV	mak2	NV	rad3	NI
ppk31	NV	lkh1	EV	fin1	EV	ppk26	NV
ppk30	EV	ppk3	EI	cki3	NI	ppk34	EV
ppk29	EV	ppk2	EV	cds1	EV	win1	EI
ppk27	EV	ppk4	EI	gsk3	NV	ppk32	EV
ppk25	EV	ppk33	EV	mik1	EV		
ppk24	EV	srk1	EV	mak1	EV		
ppk23	EV	wis4	EV	dsk1	EI		
ppk22	EV	shk2	EV	cki1	EV		
ppk20	EI	mde3	EV	cdr2	EI		
ppk19	NV	hhp2	EV	psk1	EV		
ppk16	EV	cmk2	EV	mek1	EV		
ppk14	EV	chk1	NI	kin1	EI		
ppk13	EV	pom1	NV	csk1	EV		
lsk1	EI	mph1	EV	cdr1	EV		
ppk11	NI	mak3	EV	ppk5	EV		

EI; elongated and inviable cells.

EV; elongated and viable cells.

NI; not elongated and inviable cells.

NV; not elongated and viable cells.

### Construction of gene tagging strains

C-terminal tagging of *chk1* and *cds1* with 3HA and 13myc was carried out using a PCR-based method [24]. The *hph*MX6 module was amplified using pFA6a-3HA-*hph*MX6 and pFA6a-13myc-*hph*MX6 [25] as templates together with pFA6a F and pFA6a R primers, as described in Table 2. Fragments approximately 500 bp in length were amplified using chk1t1, chk1t2, chk1t3, and chk1t4 or cds1t1, cds1t2, cds1t3, and cds1t4 primers with homology sequences corresponding to the 5′ and 3′ regions of *chk1* and *cds1* and were attached to the ends of the *hph*MX6 module. The resulting fragments were introduced into cells. Hygromycin resistant colonies were selected on YES plates containing hygromycin B (50 mg/ml). Colony PCR (using chkHR12-42, chk1R, and cds1R primers) and western blotting were performed to confirm the construction of tagging strains.

**Table 2:** *S. pombe* strains used in this study.

strain	genotype	source
L972	*h* ^−^	lab stock
SKP605-33	*h* ^+^ *leu1-32 ura4-D18 asf1-33-13myc-kan^r^*	this study
SKP593-33P	*h* ^−^ *asf1-33-13myc-kan^r^*	this study
SKP593-30	*h* ^−^ *leu1-32 ura4-D18 asf1-30-13myc-kan^r^*	lab stock
SKP561-15	*h* ^−^ *leu1-32 ura4-D18 asf1-13myc-kan^r^*	lab stock
TH1	*h* ^−^ *asf1-33-13myc-kan^r^ cds1-3HA-hph^r^ leu1-32 ura4-D18*	this study
TH9	*h* ^−^ *asf1-33-13myc-kan^r^ chk1-13myc-hph^r^*	this study
TH18	*h* ^−^ *asf1-33-13myc-kan^r^ rad3::ura4* ^+^ *ura4-D18 leu1-32 his2*	this study
TH19	*h* ^−^ *asf1-33-13myc-kan^r^ chk1::ura4* ^+^ *ura4-D18*	this study
TH20	*h^?^ asf1-33-13myc-kan^r^ cds1::ura4* ^+^ *ura4-D18*	this study
SKP551-6	*h* ^+^ *leu1-32 ura4-D18 otr1*::*ura4* ^+^	this study
SKP593-34	*h* ^+^ *leu1-32 ura4-D18 otr1*::*ura4* ^+^ *asf1-33-13myc-kan^r^*	this study
AL2768	*h^−^ leu1-32 ura4-D18 ade6-704 chk1-S345A:9myc:2HA:6His:ura4* ^+^	Paul Russell
TH34	*h* ^+^ *asf1-33-13myc-kan^r^ chk1-S345A:9myc2HA6His ade6-704*	this study
SKP558-7	*h* ^+^ *leu1-32 ura4-D18 his2 rad22-GFP-kan^r^*	this study
KT166	*h* ^−^ *rad22-GFP-kan^r^ asf1-33-13myc-kan^r^ leu1-32*	this study
HM664	*h* ^−^ *ura4::ura4* ^+^ *nmt1-TK* ^+^	Hisao Masukata
KT68	*h* ^−^ *ura4::ura4* ^+^ *nmt1-TK* ^+^ *asf1-33-13myc-kan^r^*	this study
FY14069	*h* ^+^ *ade6-M210 leu1-32 ura4-D18 tel1::ura4* ^+^	Fuyuki Ishikawa
MBY1747–MBY1844	*h* ^−^ leu1-32 ura4-D18 ppk**::ura4 ^+^	Mohan Balasubramanian

### Screening of multi-copy suppressor in the *asf1-33* mutant

SKP605-33 (*asf1-33-13myc-*kanMX6) was transformed with an *S. pombe* genomic DNA library, pTN-L1 [26], and incubated on EMM-Leu plates at 26°C. Colonies were replica-plated to YES plates containing phloxine B and cultured at 26, 34, and 36°C for 24 h. The color and morphology of cells were observed microscopically. Transformants that grew at 34 or 36°C were selected and the plasmids were extracted from them. SKP605-33 (*asf1-33* mutant) was retransformed with the candidate plasmids. The sequence of candidate plasmids was determined with a DNA sequencer (Applied Biosystems, Foster city, CA, USA).

### Cloning of *sim3* gene into pREP41 vector

The *sim3* gene was cloned into pREP41 using a gap-repair cloning method [27]. The ORF region of *sim3* containing the pREP41 recombination site homology sequence was amplified by PCR. This fragment, together with BamHI digested pREP41, was used to co-transform PR110 (h^+^ *leu1-32 ura4-D18*) and transformants were selected on EMM-Leu. The plasmids were extracted from transformants and introduced into *Escherichia coli* DH5α to amplify the plasmids. Correct construction of the plasmids was confirmed by sequencing using P_nmt1_ 80 bp F and T_nmt1_ 80 bp R primers.

### Western blotting, immunofluorescence, and immunoprecipitation

Western blotting, indirect immunofluorescence and immunoprecipitation were performed essentially as described previously [28], [29]. For immunoprecipitation, 3 µl of the anti-myc antibody (9E11, Santa Cruz Biotechnology Inc., CA, USA) was added to 100 µl of Protein G sepharose solution. Two milligrams of total protein was mixed with 100 µl bead suspension and incubated at 4°C for 1 h. Supernatants were removed after centrifugation (7,000 rpm at 4°C). Beads were washed three times with HB buffer (25 mM MOPS pH 7.2, 60 mM ß-glycerophosphate, 15 mM MgCl_2_, 15 mM EGTA, 1 mM DTT, 1% Triton X-100, and 100 mM NaCl). 6 µl 5×SDS-sample buffer was added to pellets. All samples were boiled at 100°C for 5 min. Histone H3 proteins co-immunoprecipitated with Asf1-13myc proteins were detected by western blotting using a C-terminal histone H3 antibody (Abcam Inc., Cambridge, UK).

### Detection of phosphorylation of Cds1 and Chk1

Extraction of Cds1-3HA and Chk1-13myc proteins from fission yeast strains was performed by rapid protein extraction using NaOH, as described previously [30]. For the detection of Cds1 phosphorylation, SDS-PAGE was performed using a polyacrylamide gel containing 25 µM Phos-tag™ [31] and 50 µM MnCl_2_. After electrophoresis, the gel was soaked in Transfer buffer containing 1 mM EDTA and incubated for 10 min with gentle shaking. The gel was then soaked in Transfer buffer without EDTA and incubated for 10 min with gentle shaking. After transfer of the gel to PVDF membrane, the membrane was incubated in blocking solution (0.1% TBST+5% BSA) at 4°C over night. Anti-HA monoclonal antibody (Santa Cruz Biotechnology Inc., Santa Cruz, CA, USA) was diluted with blocking solution (1∶2,000) and incubated with the membrane at room temperature for 1 h. The membrane was rinsed with 0.1% PBST three times. Anti-mouse horseradish peroxidase-conjugated antibody (Santa Cruz Biotechnology Inc., Santa Cruz, CA, USA) was diluted with blocking solution (1∶2,000) and incubated with the membrane at room temperature for 1 h. Protein bands on the membrane were detected with the ECL system (GE Healthcare). For the detection of Chk1 phosphorylation, polyacrylamide gel containing 4% acrylamide-N,N′-methylene-bis-acrylamide (acrylamide-bis) (200∶1), 70 mM Tris (pH 6.7), 4 mM EDTA and 0.4% SDS was prepared.

### Pulse Field Gel Electrophoresis

Pulse field gel electrophoresis was performed as previously described [32]. Logarithmically growing cells were incubated in YES medium at 26°C or 36°C for 6 h. Cells were collected by centrifugation and washed twice with CSE (20 mM citrate/phosphate pH 5.6, 40 mM EDTA, and 1.2 M Sorbitol). Ten milliliters of CSE (containing 15 mg Zymolyase 20T) was added to the cell pellets, followed by incubation at 37°C for 1 h. After cell permeabilization and treatment with Proteinase K, pulse field gel electrophoresis was carried out on a 0.6% chromosomal grade agarose gel (Bio Rad) with a Bio Rad CHEF-DR apparatus. The gel was run for 48 h at 50 V with a switch time of 30 min in 0.5×TAE at 14°C. The electrophoresis buffer was refreshed after 24 h. After electrophoresis, the gel was stained with ethidium bromide.

### Micrococcal nuclease digestion of chromatin

Micrococcal nuclease assay was performed as described previously [33]. Cells were incubated at 26°C or 36°C for 6 h. Chromatin was digested using micrococcal nuclease, separated by 1.2% agarose gel electrophoresis, and stained with ethidium bromide.

### Chromatin immunoprecipitation

Chromatin immunoprecipitation was performed as previously described [34], with some modifications. Immunoprecipitation was performed using an anti-H3 antibody (Abcam Inc., Cambridge, UK). Immunoprecipitated DNA was extracted and subjected to Real Time PCR analysis with centromere region-specific primers (cnt1F, cnt1R, imr1F, imr1R, dgF, dgR, dhF, and dhR) as described in Table 2.

The percentage of immunoprecipitated DNA (IP %) in ChIP samples was calculated relative to the initial amount of DNA.

### Synchronization of cell cycle and FACS analysis

Cells were incubated at 26°C in EMM2 medium containing 20 mM hydroxyurea (HU) (Sigma) for 4 h to block the progression of the cell cycle at the G1/S phase. Synchronized cells were washed with sterile water three times. Subsequently, samples were inoculated to EMM2 without HU and incubated at 26°C or 36°C for 90 min. To synchronize cell cycle progression at G1 phase, logarithmically growing cells incubated in EMM2 with nitrogen at 26°C for 12 h were washed with sterile water three times. The cells were then inoculated to nitrogen-free EMM2 medium and incubated at 26°C for 12 h to arrest cell cycle progression at G1 phase. G1 arrested cells were transferred to YES medium and cultured at 26°C or 36°C for 6 h. Samples were collected every 15 min (HU block) or 1 h (nitrogen starvation) by centrifugation. Ethanol was added to cell pellets, with vigorous vortexing. Cells were collected by centrifugation and washed once with 50 mM sodium citrate buffer (pH 7.0). RNase A was added to the samples and incubated at 37°C for 1 h. RNase A-treated samples were transferred to BD FACS flow (Becton-Dickinson) containing 20 µg/ml propidium iodide (Sigma). Cellular DNA was detected by a FACSCalibur with CELL Quest software (Becton Dickinson).

### Monitoring DNA replication by BrdU incorporation

The BrdU incorporation assay was performed as described previously [35], with some modification. Cells expressing thymidine kinase under the control of the *nmt1* promoter were incubated in EMM2 (without thiamine) for more than 12 h to induce thymidine kinase gene expression. BrdU (Sigma, B-9285) was added to the media (200 µg/ml) and the cells were incubated at 26°C or 36°C for 4 h. Cells were collected by centrifugation and fixed with ethanol for 10 min. Cells were resuspended in 1 ml of 3.5 M HCl and incubated for 10 min to denature the DNA and were then washed with PBS. The cells were then suspended in PBS containing 5% BSA and incubated at room temperature for several hours. Anti-BrdU antibody (Becton Dickinson, Lincoln Park, NJ, USA 1∶50 in PEMBAL) was added to each sample and followed by incubation at room temperature for 12 h. Cells were washed three times with PBS containing 5% BSA, and Alexa fluor 488-conjugated anti-rabbit antibody (Invitrogen, 1∶250) was added. After incubation at room temperature for several hours, cells were washed three times with PBS containing 5% BSA. Fluorescence images were taken using an Olympus BX51 fluorescence microscope system.

### Extraction of histone proteins

Extraction and analysis of histone proteins by SDS-PAGE was performed as described previously [33]. Logarithmically growing cells were incubated in YES medium at 36°C for 6 h. Cells were collected by centrifugation and histone proteins were extracted from those cells. After electrophoresis, histone proteins were visualized with Coomassie blue.

### RNA extraction and RT-qPCR analysis

Cells were cultured in YES medium at 26 or 36°C for 6 h. Cells were collected by centrifugation and pellets were suspended in 400 µl of AE buffer (50 mM sodium acetate pH 5.3, 10 mM EDTA). Then, 40 µl of 10% SDS was added to each sample, and the suspension was vortexed. 440 µl of TE-saturated phenol was added, and vortexed. The mixture was freezed at −80°C and freezed samples were incubated at 65°C for 4 min. The mixture was dipped into liquid N_2_, and freezed completely. This freeze-thaw cycle was repeated 3 times. After centrifugation at 14,000 rpm (16,000 g) for 5 min, supernatant was transferred to a new tube. Equal volume of phenol∶chloroform∶isoamylalchol was added, and centrifuged at 14,000 rpm for 5 min. Supernatant was transferred to a new tube, and 1/10 volume of 3M sodium acetate and ×2.5 volume of EtOH was added. After centrifugation at 14,000 rpm, 4°C, for 15 min, supernatant was removed. The precipitated RNAs were rinsed with 70% EtOH. Pellets were suspended in 50 µl of DEPC water. 3 µg of total RNAs were used for RT-qPCR analysis. RT-qPCR was performed with Takara one-step SYBR Prime Script RT-PCR kit (Perfect Real Time) according to the manufacturer's instruction. Quantified DNA was normalized against *act1*. Primers (act1 RT F, act1 RT R, ura4 RT F, and ura4 RT R) used are listed in Table 3.

**Table 3:** Oligonucleotides used in this study.

Name	Sequence
chk1 t1	Cttatcccagccagtacaac
chk1 t2	cgtcgacctgcagcgtacgaatttgtgaaacatctgtaagaac
chk1t	cgagctcgaattcatgatgtgcacatcttttgaaaggtcg
chk1 t4	gaagacatgttaatgtctgcag
cds1 t1	ttattcatgggacgggaacc
cds1 t2	cgtcgacctgcagcgtacgaactcgaagaattgacgtgttc
cds1 t3	cgagctcgaattcatcgatgtaaccactctgtccacgtac
cds1 t4	tttcagcgataggtttggcg
cds1-check	atcgtaagcaaagttattattgg
chk1-check	aatgaaaaggtgagtttaggag
pFA6a-F	tcgtacgctgcaggtcgacg
pFA6a-R	catcgatgaattcgagctcg
chk HR 42-14	gctaggatacagttctctcaca
cnt1 F	gtaaagtaacagatatgtttcgc
cnt1 R	gcgtttcttcggcgaaatgc
imr1 F	tcgatattactaggtgagtag
imr1 R	gctgaggctaagtatctgtt
dg F	atatccaactgacggcatcg
dg R	tcataaagcaacatgggtg
dh F	gtcgtagatgtgacgtcaac
dh R	ggaacaaatcaggaaaccgag
act1 RT F	ggtcaagattgttgctcctc
act1 RT R	cgctctcatcatactcttgc
ura4 RT F	agcaatatcgtactcctgaag
ura4 RT R	atgctgagaaagtctttgctg

## Results

### Isolation of a temperature sensitive *asf1* mutant that showed elongated cell shape

**Figure 1:**
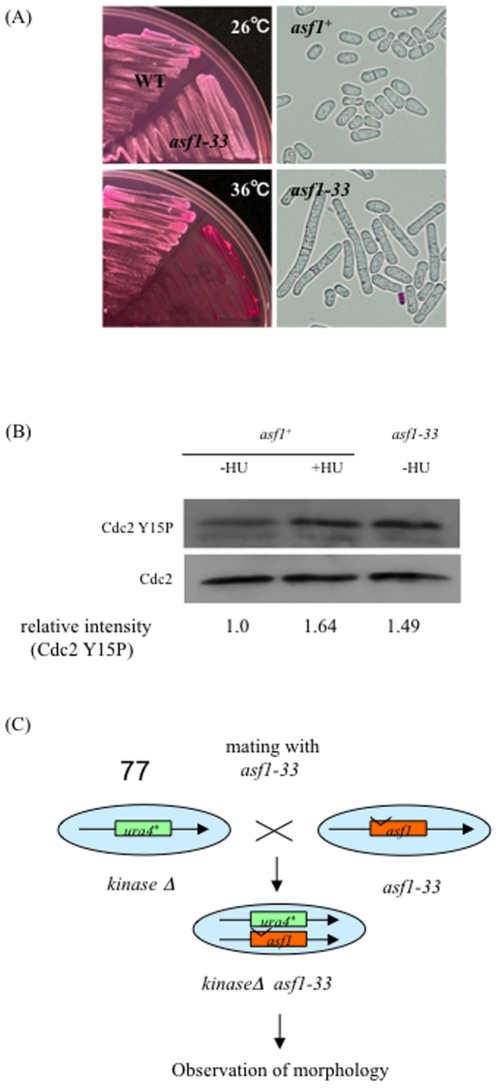
Failure to grow at 36°C and elongated cell shape in an *S. pombe asf1* mutant. (A) L972 (*asf1^+^*) and SKP605-33 (*h^−^ leu1-32 ura4-D18 asf1-33-13myc-kan^r^*) were grown on YES plates containing phloxine B at 26°C or 36°C for 24 h. Cell morphology was observed by a microscope. (B) Cdc2 (Y15) was highly phosphorylated in SKP605-33 (*asf1-33*-*13myc*-*kan^r^*) at the restrictive temperature. L972 (*asf1^+^*) and SKP605-33 (*asf1-33*-*13myc*-*kan^r^*) were incubated at 26°C or 36°C for 6 h. Cells were collected by centrifugation and washed once with STOP buffer. Protein extraction was performed by the glass-beads method. Samples were suspended into SDS-sample buffer. Ten micrograms of total proteins was used for western blotting. The relative intensity of each band (Cdc2Y15P) relative to the control (Cdc2) was measured using ImageJ (http://rsb.info.nih.gov/ij/). (C) Schematic representation of the strategy to identify protein kinases responsible for activation of the cell cycle checkpoint in the *asf1-33* mutant.

To investigate the functions of *asf1*, we constructed *asf1* temperature sensitive mutants because *asf1* is essential for growth in *S. pombe* [21]. Mutations were randomly introduced into the *asf1* gene by an error-prone PCR method, and PCR products linked to a *kan* marker gene were inserted into the chromosomal locus of *asf1*. We then selected temperature sensitive mutants that could hardly grow at 36°C [29]. Some *asf1*-ts mutants showed elongated cell shape, which suggested their cell cycle is delayed or arrested. The *asf1-33* mutant, which had the longest cell shape at the restrictive temperature (Fig. 1A), was selected for further analysis. Sequencing of the *asf1-33* allele revealed that it contained six missense mutations that resulted in amino acid substitutions A16T, L61P, E119K, L121P, N155S, and E180G.

The phenotype of the *asf1-33* mutant led us to test the phosphorylation of Cdc2 because Cdc2 is phosphorylated at Y15 when the cell cycle is arrested [36], [37]. Phosphorylation decreases the activity of Cdc2, which is followed by cell cycle arrest at G2/M phase. Cdc2 phosphorylation was detected with a specific antibody, Cdc2Y15P. Increased Cdc2 phosphorylation was detected in the *asf1-33* mutant compared to the *asf1^+^* strain (Fig. 1B), which suggests that the cell cycle is delayed or arrested and that the checkpoint might be activated in the *asf1-33* mutant at 36°C.

### The *asf1-33* mutant required Rad3 and Chk1 kinases for cell cycle arrest and survival

The cell elongation phenotype and phosphorylation of Cdc2 led us to test checkpoint activation in the *asf1-33* mutant. As cell cycle checkpoint pathways frequently consist of a set of protein kinases [36], we considered the possible involvement of novel protein kinases. To that end, we used a deletion set of protein kinases constructed by M. Balasubramanian [38] to generate double mutants with *asf1-33* mutant by mating (Fig. 1C). Some deletion strains that were deficient in MAP kinases were excluded from this experiment because MAP kinases are unlikely to be involved in the mitotic cell cycle checkpoint [39]. Strains lacking 77 different kinases were mated with the *asf1-33* mutant on nitrogen-free EMM2 to construct double mutants. Random spore analysis was performed, and double mutants lacking a specific kinase gene and possessing the *asf1-33* mutation were selected by G418 resistance and uracil auxotrophy. Cell morphology and viability of the 77 strains was examined after incubation at 36°C for 24 h on YES medium (Table 3). The phenotypes of each double mutant were categorized into four types: (1) not elongated and enhanced lethality, (2) not elongated and retained viability, (3) elongated and enhanced lethality, and (4) elongated and retained viability. Since the *asf1-33* mutant was still able to grow slowly at the restrictive temperature, we sought to identify the protein kinase deletion mutants that lost their viability at 36°C. Double mutants that lost the cell elongation phenotype of the *asf1-33* mutant were also selected. We considered that the viability and morphology were critical to identify checkpoint kinases operative in the *asf1-33* mutant.

Of the 77 kinases tested, we found that the deletion of *chk1* and *rad3*, when combined with the *asf1-33* mutation, caused severe defects in cell elongation and resulted in enhanced cell death. Because Chk1 is controlled by Rad3-mediated phosphorylation in response to DNA damage [40], the results highlight the significance of DNA damage checkpoint factors for the function of *asf1-33*. We also examined whether the deletion of *tel1*, which encodes a homologue of ATM checkpoint kinase and was not included in the deletion set of protein kinases, affects the growth of the *asf1-33* mutant at 36°C. However, the growth of the *asf1-33 Δtel1* double mutant was similar to the *asf1-33* mutant, indicating that *tel1* did not confer a synthetic effect in the *asf1-33* mutant at 36°C (data not shown).

### Chk1 checkpoint pathway is activated in the *asf1-33* mutant

**Figure 2:**
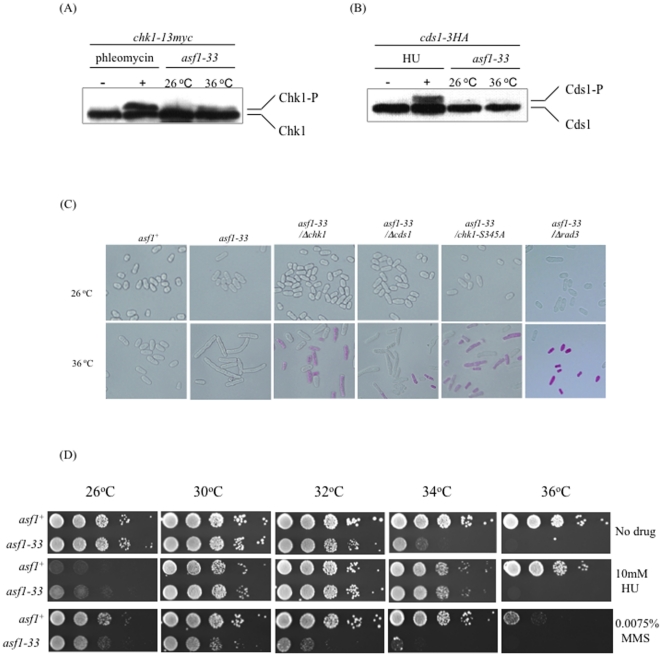
DNA damage checkpoint was activated in the *asf1-33* mutant at 36°C. (A&B) Chk1, but not Cds1, was phosphorylated in the *asf1-33* mutant at 36°C. Phosphorylation of Chk1 and Cds1 proteins was observed by the mobility shift of phosphorylated proteins during electrophoresis. TH1 (*asf1-33*-*13myc*-*kan^r^ cds1^−^-3HA*) and TH9 (*asf1-33*-*13myc*-*kan^r^ chk1^−^-13myc*) were incubated in YES medium at 26°C and 36°C for 6 h. 30 µg and 15 µg of total proteins were used for detecting Chk1 and Cds1, respectively, by western blotting. HU is used as a DNA replication inhibitor which arrests cell cycle progression at G1/S phase. MMS is used as a DNA damaging agent. Addition of HU and MMS induced mobility shift of phosphorylated Cds1 and Chk1 proteins, respectively. (C) L972 (*asf1^+^*), SKP593-33P (*asf1-33*-*13myc*-*kan^r^*), TH19 (*asf1-33*-*13myc*-*kan^r^ Δchk1* mutant), TH20 (*asf1-33*-*13myc*-*kan^r^* Δ*cds1* mutant), and TH34 (*asf1-33*-*13myc*-*kan^r^ chk1S345A* mutant) were grown on YES plates containing phloxine B at 26°C and 36°C for 24 h. Cell morphology was observed by a microscope. (D) Cultures of L972 (*asf1^+^*) and SKP605-33 (*asf1-33*-*13myc*-*kan^r^*) were serially diluted with sterilized water. The cells were spotted on YES plates containing 10 mM HU (DNA replication inhibitor) and 0.0075% MMS (DNA damaging agent) and cultured at respective temperature for 3 days.

The DNA damage checkpoint is activated in response to exogenous or endogenous DNA damage and protects genomic DNA [40]. The sensor kinase Rad3 detects DNA damage in chromatin and transduces a signal to an effecter kinase, Chk1, by phosphorylating it [41]. The requirement for *rad3* and *chk1* for the survival of the *asf1-33* mutant suggested that the Chk1 pathway was activated in these cells. We therefore examined whether Chk1 is phosphorylated in the *asf1-33* mutant at 36°C by testing for a phosphorylation-induced mobility shift in Chk1 using phosphate-binding tag (Phos-tag™ AAL-107) in a phosphate affinity SDS-PAGE. In this assay, phosphorylated proteins are captured by Phos-tag™ in the SDS-PAGE gel during electrophoresis and their mobility is super-shifted. Using this method, phosphorylated-Cds1 protein was identified but there was no evidence for Chk1 phosphorylation. We then changed the acrylamide∶bisacrylamide ratio from 37.5∶1 to 200∶1 in order to more clearly separate phosphorylated and non-phosphorylated Chk1. Using these conditions, we were able to detect the mobility shift of phosphorylated Chk1 in the *asf1-33* mutant at 36°C by western blotting (Fig. 2A). In contrast, Cds1, a DNA replication checkpoint factor, was not phosphorylated in the *asf1-33* mutant at 36°C (Fig. 2B). Furthermore, we found that a phosphorylation-deficient mutant of *chk1* (*chk1* S345A) [42] showed a similar phenotype to the *asf1-33 Δchk1* mutant (Fig. 2C). Taken together, these results indicated that a DNA damage checkpoint, but not a DNA replication checkpoint, was activated in the *asf1-33* mutant at 36°C.

We next examined the drug sensitivity of the *asf1-33* mutant at different temperatures. At the semi-restrictive temperature (34°C), the *asf1-33* mutant was sensitive to the DNA damaging agent methyl methanesulfonate (MMS) (Fig. 2D). This result is consistent with the requirement of DNA damage checkpoint factors for survival and cell cycle checkpoint activation in the *asf1-33* mutant. In contrast, the *asf1-33* mutant was not sensitive to hydroxyurea (HU) at 34°C (Fig. 2D). This result is consistent with the result that the *asf1-33* mutant did not require *cds1*, which encodes a DNA replication checkpoint factor.

### Asf1 was required for the maintenance of genomic stability

**Figure 3:**
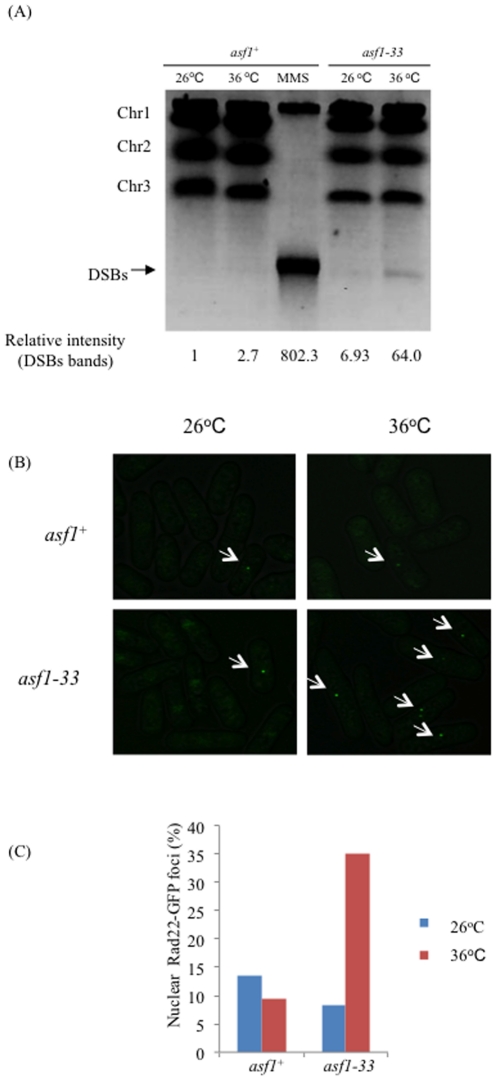
DNA double-stranded breaks occurred in the *asf1-33* mutant. (A) Pulse-field gel electrophoresis analysis of the *asf1-33* mutant. L972 (*asf1^+^*) and SKP605-33 (*asf1-33*-*13myc*-*kan^r^*) were incubated in YES medium at 26°C and 36°C for 6 h, and 5.0×10^8^ cells were collected by centrifugation. Pulse field gel electrophoresis was performed as described in the Materials and Methods. The intensity of DSB bands was measured using ImageJ. (B) Rad22-GFP foci in SKP558-7 (*asf1^−^*) and KT166 (*asf1-33*-*13myc*-*kan^r^*) were observed after incubation at 26°C or 36°C for 6 h. Fluorescence of Rad22-GFP foci was observed with a Leica TCS-SP5 confocal laser scanning microscope (Leica Microsystems, Japan). Arrows indicate the Rad22-GFP foci. (C) The number of Rad22-GFP foci was counted.

The phosphorylation of Chk1 in the *asf1-33* mutant (Fig. 2B) suggested that DNA damage occurred in these cells. We therefore tested for DNA double-strand breaks using pulse-field gel electrophoresis. Partial but detectable DNA double-stranded breaks occurred in the *asf1-33* mutant at 36°C (Fig. 3A). Although the amount of DNA damage in the *asf1-33* mutant was not great, it was sufficient to activate the DNA damage checkpoint, as shown in Fig. 2. The result also suggested that the cell cycle is not arrested during S phase in the *asf1-33* mutant at 36°C because three chromosomes entered the gel without any remaining DNAs in the wells. We next tested whether Rad22-GFP foci are formed in the *asf1-33* mutant. Fission yeast Rad22 is a DNA repair protein required for homologous recombination. In response to DNA damage, Rad22 accumulates at the sites of damage and forms foci [43]. A significantly higher level of Rad22-GFP foci was detected in the *asf1-33* mutant at 36°C than in the *asf1^+^* strain (Fig. 3B,C). This result further indicated that DNA damage occurred in the *asf1-33* mutant at 36°C.

### S phase progression was not delayed in the *asf1-33* mutant

**Figure 4:**
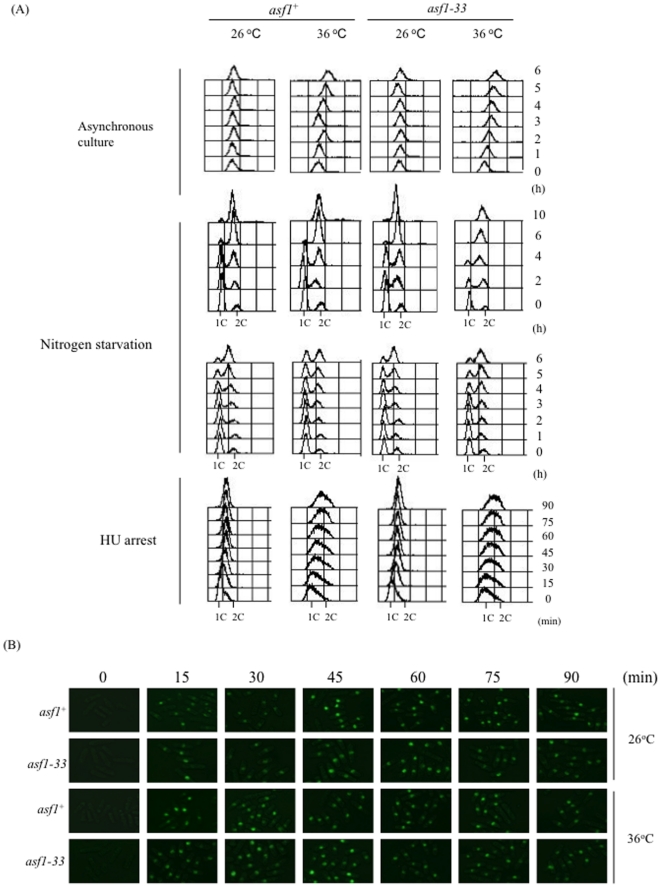
Cell cycle progression was not delayed in *asf1-33* mutant. (A) Cell cycle progression of L972 (*asf1^+^*) and SKP593-33P (*asf1-33*-*13myc*-*kan^r^*) was blocked at G1/S phase by incubation in EMM2 medium containing 20 mM hyroxyurea at 26°C for 4 h. To synchronize cell cycle progression, cells were incubated in EMM (-Nitrogen) at 26°C for 12 h to induce G1 arrest. The cells were washed three times with sterilized water and then incubated in EMM2 medium at 26°C or 36°C for 90 min (HU block & release) or in YES medium for 6 h (G1 arrest & release). Samples for FACS analysis were obtained every 15 min (for HU block and release) or 2 h (for G1 arrest and release). Cells were fixed with ethanol and stained with propidium iodide after RNase A treatment. (B) BrdU incorporation at S phase was not decreased in the *asf1-33* mutant at 36°C. HM664 (*nmt1-TK^−^*) and KT68 (*asf1-33*-*13myc*-*kan^r^ nmt1-TK^−^*) were incubated in EMM (without thiamine) at 26°C for more than 12 h to induce thymidine kinase gene expression. The cells were transferred to EMM containing 200 µg/ml BrdU and incubated for 90 min at 26°C or 36°C. Samples were collected by centrifugation every 15 min and fixed with ethanol. The cells were treated with Zymolyase to make spheroplasts. After washing with PBS, 3.5 M HCl was added to samples to denature the DNA. Anti-BrdU antibody was added and the samples were incubated at 26°C for 24 h followed by incubation with anti-mouse IgG Alexa 488-conjugated antibody. Fluorescence images were taken with a fluorescence microscope.

Asf1 incorporates histones H3/H4 onto nascent DNA strands during S phase in cooperation with CAF1 [44]. Therefore, we considered the possibility that the loss of Asf1-33 function might influence the progression of S phase in the *asf1-33* mutant at 36°C. Cell cycle progression in the *asf1-33* mutant was monitored by FACS analysis. However, following synchronization of the cell cycle by either nitrogen starvation or HU block, cell cycle progression from the G1 phase was not delayed in the *asf1-33* mutant (Fig. 4A).

We next tested the progress of DNA replication by measuring the incorporation of bromodeoxyuridine (BrdU) into replicating DNA strands. To that end, we created a strain that expresses thymidine kinase because this enzyme is absent in *S. pombe* but is required for the incorporation of BrdU [45]. We constructed *asf1-33 nmt1-TK* ^+^; this strain was synchronized at G1/S phase with HU and after removal of HU was incubated at 26°C or 36°C for 90 min in YES medium containing 200 mg/ml BrdU. Most cells incorporated BrdU within 15 minutes after release from HU block (Fig. 4B). These results showed that cell cycle progression during S phase was not delayed in the *asf1-33* mutant.

### Binding of Asf1-33 with Histone H3 and Localization of Asf1-33 protein

**Figure 5:**
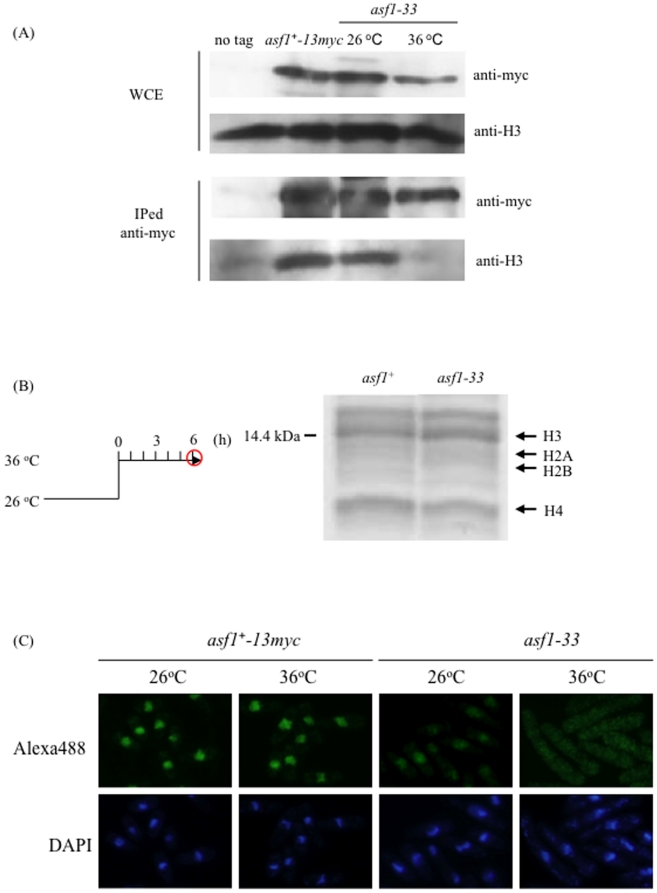
Interaction between Asf1 and histone H3 was lost and Asf1-33 proteins were mislocalized at 36°C in the *asf1-33* mutant. (A) Immunoprecipitation assay to investigate the interaction between Asf1 and histone H3. L972 (*asf1^+^*), SKP561-15 (*asf1^−^-13myc-kan^r^*) and SKP605-33 (*asf1-33-13myc-kan^r^*) strains were incubated at 26°C and 36°C for 6 h. The cells were collected by centrifugation and washed once with STOP buffer. Two milligrams of total proteins were used. After incubation with Protein G sepharose at 4°C for 1 h, the samples were washed five times with HB buffer. The samples were suspended in SDS-sample buffer and boiled at 100°C. Proteins were detected by western blotting as described in Materials and Methods. (B) Extraction of histone proteins from *asf1* mutants. L972 (*asf1^+^*) and SKP593-33P (*asf1-33-13myc-kan* ^r^) strains were incubated at 26°C for 24 h. The temperature was increased to 36°C and cells were incubated for a further 6 h. Extraction of histone proteins was performed as described in the Materials and Methods. Extracted histone proteins were separated by SDS-PAGE and stained with Coomassie Blue. (C) Immunofluorescence images showing the localization of Asf1-13myc in the *asf1^+^* strain and *asf1-33* mutant. SKP561-15 (*asf1^+^-13myc-kan^r^*) and SKP605-33 (*asf1-33*-*13myc*-*kan^r^*) were incubated at 26°C or 36°C for 6 h and immunofluorescence analysis was performed as described in Materials and Methods.

We next examined whether Asf1-33 binds to histone H3 at 36°C. Wild-type Asf1 (at 26°C or 36°C) and Asf1-33 (at 26°C) were co-immunoprecipitated with histone H3, but Asf1-33 did not co-immunoprecipitate with histone H3 at 36°C (Fig. 5A). The level of histone proteins in the *asf1-33* mutant and *asf1^+^* cells was indistinguishable, confirming the mutations of *asf1* do not affect histone levels in fission yeast but do lead to alterations in histone H3 binding (Fig. 5B).

We next observed the cellular localization of Asf1-33. Immunofluorescence using an anti-Myc antibody showed mislocalization of Asf1-33-13myc at 36°C. Wild-type Asf1-13myc (at 26°C or 36°C) and Asf1-33-13myc at 26°C were in the nucleus, but at 36°C Asf1-33 was seen throughout the cytoplasm (Fig. 5C).

### *asf1-33* mutations cause drastic defects in chromatin structure

**Figure 6:**
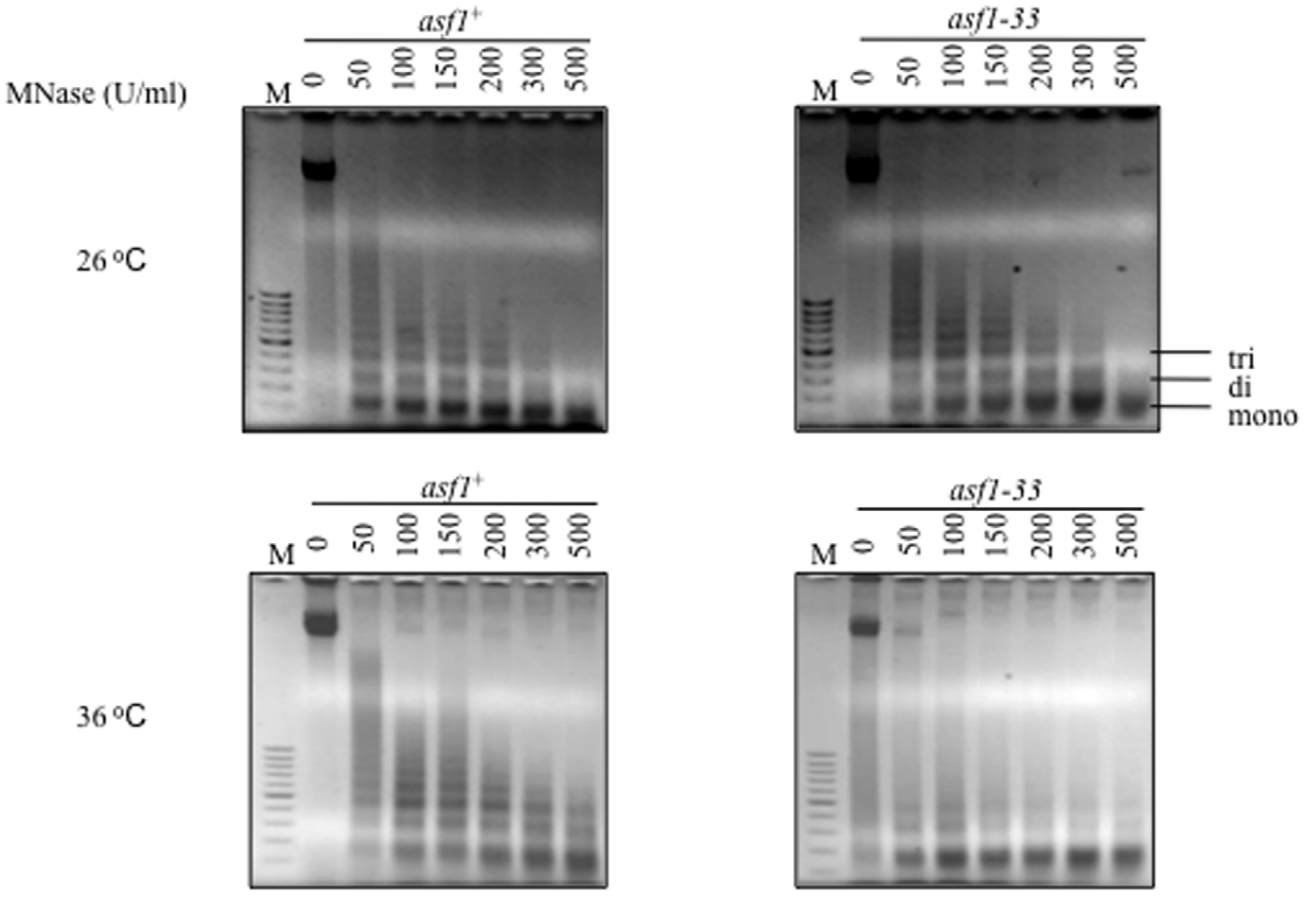
*asf1* mutations caused defects in chromatin structure at 36°C. L972 (*asf1^+^*) and SKP593-33P (*asf1-33*-*13myc*-*kan^r^*) were treated with Zymolyase to make spheroplasts. MNase was added to the spheroplasts and incubated at 37°C for 5 min. Digested chromatin DNA was resolved by agarose gel electrophoresis and detected with ethidium bromide staining.

Asf1 is involved in chromatin assembly and disassembly through binding to histones H3/H4. Since the binding of Asf1-33 to histone H3 was impaired, we tested chromatin structure in the *asf1-33* mutant using MNase (Micrococcal Nuclease). MNase cuts the linker regions of chromatin DNA, and the digested chromatin DNAs are separated by agarose gel electrophoresis, with the resulting ladder pattern reflecting the chromatin structure. When we performed a MNase assay for the *asf1-33* mutant, no significant changes in chromatin structure were observed in the *asf1-33* mutant at 26°C, but the ladder pattern was different at 36°C, with a strong accumulation of mono nucleosomes (Fig. 6). This result suggested that lethality in the *asf1-33* mutant may be related to defects in chromatin structure [46].

### Impaired transcriptional silencing due to the *asf1-33* mutation

**Figure 7:**
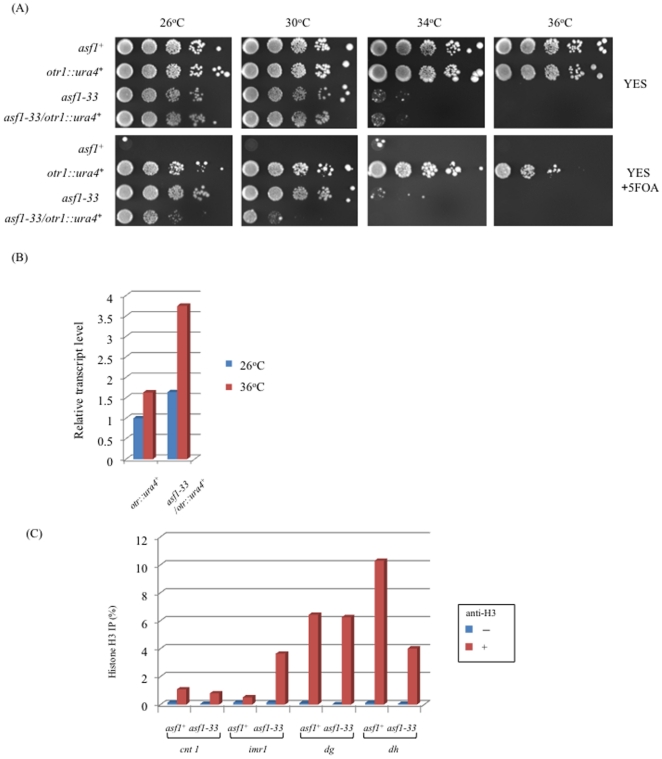
Histone loading in the *asf1-33* mutant and heterochromatic silencing at the outer centromeric repeat. (A) Cultures of SKP551-6 (*otr1*::*ura4^−^*) and SKP593-33 (*asf1-33*-*13myc*-*kan^r^ otr1*::*ura4^+^*) were subjected to serial dilution with sterilized water and spotted on YES plates containing 5-FOA. Each strain was incubated at 26, 34, and 36°C for several days. (B) RT-qPCR analysis of the *asf1-33* mutant. L972 (*asf1^+^*) and SKP605-33 (*asf1-33*-*13myc*-*kan^r^*) were cultured in YES medium at 26°C or 36°C for 6 h. After incubation, cellular RNA was extracted as described in Materials and Methods. RT-qPCR was performed by using primer sets amplifying *ura4* and *act1*. (C) ChIP analysis of the *asf1-33* mutant. L972 (*asf1^+^*) and SKP605-33 (*asf1-33*-*13myc*-*kan^r^*) were incubated in YES medium at 36°C for 6 h and the cells were collected by centrifugation. Immunoprecipitation was performed using an anti-C terminal H3 antibody. After immunoprecipitation, DNA was extracted and amplified by PCR with specific primers for quantitative analysis.

Heterochromatin is composed of condensed chromatin, which is transcriptionally silent. The *S. pombe* centromere is divided into two transcriptionally silent domains: the central core domain in which kinetochore chromatin is assembled and the heterochromatic outer centromeric domain [47]. Histone chaperones are involved in the maintenance of heterochromatin structure and its transcriptional silencing [48], [49]. To determine whether *asf1* is required for transcriptional silencing at the centromeric outer repeat region, we examined the expression of a reporter gene inserted at the outer repeat domain of the centromere. Expression of the *ura4* ^+^ gene located in the outer repeat of the centromere is normally repressed and wild-type cells do not show sensitivity to 5-FOA. When heterochromatin structure is disrupted, the expression of the *ura4* ^+^ gene is derepressed and the cells become sensitive to 5-FOA. [50]. The *asf1-33* mutation caused sensitivity to 5-FOA in the strain with the *ura4* ^+^ gene integrated at the outer centromeric repeat (*otr*) (Fig. 7A). In addition, we measured the transcription level of the *ura4* ^+^ gene at the centromere in the *asf1-33* mutant by RT-qPCR and found that it was increased at 36°C than at 26°C (Fig. 7B). These results suggested that *asf1* is required for the maintenance of heterochromatin structure in fission yeast. These results led us to test the histone H3 levels at the centromere region in the *asf1-33* mutant by ChIP analysis. We found that histone H3 levels at the outer repeat (*dh*) of the centromere heterochromatic region were decreased in the *asf1-33* mutant (Fig. 7C). The chromatin assembly activity of Asf1 seems to be necessary for the maintenance of the centromere heterochromatic region in fission yeast. Interestingly, histone H3 levels at the central centromeric region (*imr1*) were increased in the *asf1-33* mutant.

### The CenH3 histone chaperone Sim3 suppresses the temperature sensitivity of the *asf1-33* mutant

To further understand the function of Asf1, we screened for multi-copy suppressors in the *asf1-33* mutant using a plasmid-borne genomic DNA library. The *asf1-33* mutants harboring genomic DNA libraries (pTN-L1) were replica-plated to YES plates containing phloxine B and incubated at 36°C for 24 h. Phloxine B stained dead cells a much darker red color than viable cells. Based on colony color and cell morphology we selected several strains that grew better at 36°C.

**Figure 8:**
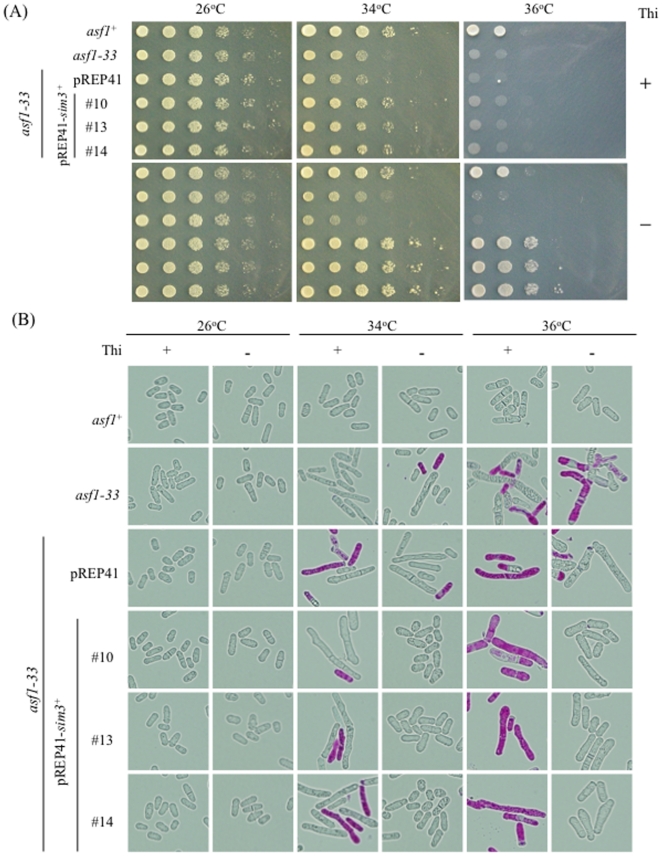
Overexpression of *sim3* reversed the temperature sensitive growth in the *asf1-33* mutant. (A) Cultures of L972 (*asf1^+^*), SKP605-33 (*asf1-33*-*13myc*-*kan^r^*), and SKP605-33 harboring pREP41 or pREP41-*sim3* were serially diluted with sterilized water and spotted on YES plates. Plates were incubated at 26, 34 and 36°C. (B) Cell morphology of the cells used in (A) was observed microscopically.

Plasmid(s) were once lost to examine whether the suppression of temperature sensitivity was dependent on the plasmid. The plasmids were then restored in *E. coli* and the *asf1-33* mutant was retransformed with candidate plasmids to confirm the reversal of temperature sensitivity. Subsequently, the gene contained within the plasmid was sequenced. In addition to *asf1*, we also isolated *sim3*, which encodes a CENP-A histone chaperone [51]. Because the library contains other genes, we cloned *sim3* into a promoter-regulated plasmid, pREP41, to confirm suppression in the *asf1-33* mutant [52]. pREP41 contains *nmt41* promoter, an attenuated version of *nmt1* promoter [53]. Promoter activity of *nmt41* is down regulated by the presence of thiamine. Overexpression of *sim3* under thiamine-depleted conditions reversed the temperature sensitivity of the *asf1-33* mutant at 36°C (Fig. 8A). In addition, the elongated cell phenotype in the *asf1-33* mutant at the restrictive temperature was reversed (Fig. 8B). This clear suppression indicated that Sim3 can replace the function of Asf1 and suggests that Sim3 may have a general role as a histone H3 chaperone in fission yeast.

## Discussion

In this study, we show that the histone chaperone Asf1 is required for the maintenance of genome stability in *S. pombe*. The *asf1-33* (ts) mutation caused a defect in chromatin structure and led to DNA damage, including double-strand breaks at a restricted temperature (Figs. 3, 5 and 6), which result in the activation of the DNA damage checkpoint (Fig. 2). By screening 77 protein kinase genes, we identified DNA damage checkpoint kinases (Chk1 and Rad3) as necessary for the survival of the *asf1-33* mutant at the restrictive temperature. The temperature-sensitive growth of the *asf1-33* mutant was further reduced and cell elongation was abolished by the deletion of *chk1* or *rad3* in the *asf1-33* mutant. We also observed that Chk1, but not Cds1, was phosphorylated in the *S. pombe asf1-33* mutant, indicating that Chk1 activation is required for the survival of the *asf1-33* mutant. In *S. cerevisiae*, the deletion of *asf1* causes DNA damage and induces phosphorylation of Rad53, which is a homolog of *S. pombe* Cds1 [54] and functions as a DNA damage checkpoint regulator. Since the DNA damage checkpoint is largely controlled by Rad53 in *S. cerevisiae*, this suggests a common role for Asf1 in protecting against DNA damage in both *S. pombe* and *S. cerevisiae*. However, while the deletion of *ASF1* causes sensitivity to the DNA replication inhibitor HU in *S. cerevisiae* [55], we did not observe this in *S. pombe* and also did not detect the phosphorylation of Cds1 in the *asf1-33* mutant at 36°C (Fig. 2). These results suggest that either Asf1 does not contribute to S phase progression or that the *asf1-33* mutant does not cause a severe defect in S phase due to a property of the specific mutation. The recent report by Yamane et al. [22] showed that the *asf1-1* mutant is sensitive to reagents that cause DNA damage but not to HU. Therefore, no requirement for Asf1 in DNA replication was observed in two independently isolated *S. pombe asf1* mutants, suggesting that Asf1 does not have a major role in S phase in *S. pombe*. In contrast to these observations in *S. pombe*, the knock-down of *asf1* in human cells [8] and in chicken DT40 cells [56] caused delayed progression of cell cycle during S phase, and similar results have been reported in *Drosophila melanogaster* [19]. Based on these reports, Asf1 is generally considered to function to incorporate histones H3/H4 into newly replicated DNA during S phase. Since Asf1 is essential for growth and our analyses were based on *asf1*-ts mutants, it is also possible that a null mutation of Asf1 might be necessary to detect its role in S-phase in *S. pombe*.

Micrococcal nuclease assay revealed that bulk chromatin structure was altered in the *asf1-33* mutant at 36°C (Fig. 6). Large changes in bulk chromatin structure by the depletion of histone H4 are lethal in *S. cerevisiae* [57], and the low viability of the *S. pombe asf1-33* mutant at 36°C might be attributed to large changes in bulk chromatin structure. In contrast to disassembled chromatin structure in the *S. pombe asf1-33* mutant, the deletion of *ASF1* in *S. cerevisiae* over-assembles its chromatin but did not cause lethality [46]. Asf1 seems to play an opposite role on chromatin structure change in fission yeast and budding yeast. Although chromatin structure change was extensive in *asf1-33* mutant, that was small in the *S. cerevisiae asf1* mutant.. This difference might reflect the specific roles of Asf1 in chromatin assembly (or disassembly) in *S. pombe*. In *S. cerevisiae*, many histone chaperones, including Asf1, CAF1, and HIRA, are cooperatively involved in changes in chromatin structure. Therefore, the deletion of *ASF1* alone may not result in severe defects in chromatin structure in *S. cerevisiae*. In contrast, in *S. pombe*, Asf1 seems to play an essential role in chromatin structure change as a histone chaperone, and roles for CAF1 and HIRA in overall chromatin structure must be limited as judged from the phenotypic analyses in these deletion mutants [22].. These differences could explain why *asf1* mutations caused severe defects in chromatin structure in *S. pombe*.

Asf1-33-13myc was mislocalized and Asf1-33-13myc could not bind histone H3 at 36°C (Fig. 5). The mutations identified in Asf1-33, which are thought to affect interactions with H3, are located within the H3 recognition region of the protein [10], [11]. Therefore, it is conceivable that an inability to bind histone H3 causes mislocalization of Asf1-33, and that the impaired histone H3 chaperone activity of Asf1 resulted in altered chromatin structure in the *asf1-33* mutant.

A silencing defect at the outer centromeric repeat was observed in the *asf1-33* mutant (Fig. 7), which is consistent with the results of Yamane et a. [22]. ChIP analysis revealed that histone H3 levels were decreased at the outer centromeric repeat (*dh*) in the *asf1-33* mutant compared to the *asf1^+^* strain (Fig. 7). However, histone H3 levels were increased at the center region of the centromere (*imr1*) in this mutant. This suggests that, in fission yeast, Asf1 functions as a chromatin assembly factor at the outer centromeric repeat (*dh*) but as a disassembly factor at the center region of the centromere (*imr1*). Disassembly of histone H3 at the center region may be required for the exchange of histone H3 and centromere-specific histone H3 variant CENP-A in *S. pombe*. Dunleavy et al. reported that histone H3 levels were increased at the center region of the centromere in *sim3* (coding a CENP-A histone chaperone) mutants [51]. This suggests that Asf1 might remove histone H3 from the center region cooperatively with Sim3.

Yamane et al. [22] showed that *S. pombe asf1-1* mutation, which abolished the binding of ASF1 to histones H3/H4, caused a defect in heterochromatic silencing and genomic instability. Although the *asf1* mutants were created independently by us and by Yamane et al, there are essentially no discrepancies between the two studies. As both Asf1 mutant proteins failed to interact with histone H3, it is reasonable that similar phenotypes were observed. In addition to their findings on heterochromatic silencing, we extended the analysis in more detail. We showed that *asf1* mutations caused a defect in chromatin structure by showing an altered micrococcal nuclease digestion pattern and we also showed the activation of the DNA damage checkpoint pathway in the *asf1-33* mutant. Activation of the DNA damage checkpoint (Fig. 2) strongly supports the idea that Asf1 plays an essential role in the maintenance of genomic stability in *S. pombe*. We also found that Asf1-33 mutant proteins were mislocalized at 36°C (Fig. 5), and proper localization of Asf1 may be important for its function. Moreover, the *asf1-33* mutant did not require S phase checkpoint factor for its survival (Fig. 2), and progression of DNA replication was not affected by the *asf1-33* mutation (Fig. 4). Unlike the results for other species, Asf1 is not essential during DNA replication in *S. pombe*. Finally, we found that high copy *sim3* suppressed the temperature sensitivity of the *asf1-33* mutant. Sim3 is an H3-like CENP-A chaperone that mainly functions to deposit CENP-A at centromeres [51]. Our results showing that Sim3 can replace the function of Asf1 provides genetic evidence that Sim3 has general roles as a histone H3 chaperone in fission yeast, which is consistent with a previous report that Sim3 binds to histone H3 [51]. It is interesting to note that the three-dimensional structure of Asf1 and the predicted structure of Sim3 do not resemble each other [51], [58] and that functional similarity between Asf1 and centromere chromatin assembly factors has not been reported in other species. Analysis of the interrelationship between these two histone H3 chaperones is an interesting subject.

Histone H3 K56 is acetylated by histone acetyl-transferase Rtt109 by forming a complex with Asf1 (or Vps75) and deacetylated by Hst3/Hst4 deacetylase in *S. cerevisiae* [59], [60]. An *in vitro* experiment showed that *S. pombe* Rtt109 homolog displays an Asf1-dependent H3 K56 histone acetyl-transferase activity [61]. An *S. pombe hst4* deletion strain showed sensitivity to DNA damaging agent [62]. These results indicated proper regulation of H3 K56 acetylation is important for maintenance of genomic stability in both *S. cerevisiae* and *S. pombe*. But an *in vivo* role of Asf1 on histone acetyl-transferase in *S. pombe* remains to be elucidated.
